# A longitudinal evaluation of free will related cognitions in obsessive–compulsive disorder

**DOI:** 10.1186/s12888-022-04108-6

**Published:** 2022-07-13

**Authors:** Maria E. Moreira-de-Oliveira, Gabriela B. de Menezes, Luana D. Laurito, Carla P. Loureiro, Samara dos Santos-Ribeiro, Leonardo F. Fontenelle

**Affiliations:** 1 D’Or Institute for Research and Education, Rua Diniz Cordeiro, 30, Rio de Janeiro, Botafogo 22281-100 Brazil; 2 Obsessive, Compulsive, and Anxiety Spectrum Research Program, Institute of Psychiatry, Federal University of Rio de Janeiro, Rio de Janeiro, Brazil; 3 Department of Psychiatry, Monash University, Clayton, VIC Australia

**Keywords:** Obsessive–Compulsive Disorder, Free Will, FWI, SAPF

## Abstract

**Background:**

Individuals with obsessive–compulsive disorder (OCD) often feel compelled to perform (compulsive) behaviors, thus raising questions regarding their free will beliefs and experiences. In the present study, we investigated if free will related cognitions (free will beliefs or experiences) differed between OCD patients and healthy subjects and whether these cognitions predicted symptom changes after a one-year follow up.

**Methods:**

Sixty OCD outpatients were assessed for their beliefs in and experiences of free will at baseline and after one year of treatment. A subsample of 18 OCD patients had their beliefs compared to 18 age and gender matched healthy controls. A regression analysis was performed to investigate whether free will cognitions at baseline were able to predict long-term OCD severity scores.

**Results:**

Patients with OCD and healthy controls do not seem to differ in terms of their beliefs in free will (U = 156.0; *p* = 0.864). Nonetheless, we found significant negative correlation between (i) duration of illness and strength of belief in determinism (*ρ* = -0.317; *p* = 0.016), (ii) age and perception of having alternative possibilities (*ρ* = -0.275; *p* = 0.038), and (iii) symptoms’ severity and perception of having alternative possibilities (*ρ* = -0.415; *p* = 0.001). On the other hand, the experience of being an owner of ones’ actions was positive correlated with the severity of symptoms (*ρ* = 0.538; *p* < 0.001) and were able to predict the severity of OCD symptoms at the follow up assessment.

**Conclusions:**

Older individuals or those with a greater severity of symptoms seem to have a perception of decreased free will. In addition, patients with a longer duration of illness tend to have a lower strength of belief in determinism. Finally, the experience of being the owner of the compulsions, along with the baseline severity of symptoms, can be a predictor of a worse outcome in the OCD sample.

**Supplementary Information:**

The online version contains supplementary material available at 10.1186/s12888-022-04108-6.

## Background

Obsessive–compulsive disorder (OCD) is characterized by recurrent and persistent thoughts, urges or images that are experienced as intrusive and unwanted (obsessions) and/or repetitive behaviors or mental acts that the individual feels driven to perform according to certain rules (compulsions) [1]. Symptoms of OCD tend to relate to three broad phenotypes, which include taboo thoughts (with aggressive, sexual or religious themes) and checking, contamination and washing, and symmetry and ordering [2]. Evidence indicates that OCD may be found in up to 3% of the general population and that it may be potentially devastating for both patients’ and their families’ quality of life [3, 4].

Despite its prevalence and far-reaching consequences, attempts to describe and understand the basic phenomenological features that characterize OCD have proved to be challenging. From a purely descriptive standpoint, the experience of “subjective compulsion” has been suggested to be OCD’s critical feature by classical psychopathologists (e.g. [5]). More recently, a Delphi review identified a few constructs thought to be shared by OCD and its related disorders, including deficits in response inhibition, problems in habit formation and “compulsivity” [6]. Although the exact definition of “compulsivity” remains elusive, it is said to be present when “the patient feels compelled to think a specific thought or to perform a specific act” [7].

The need to do something suggests that free will may play an important role in the OCD phenomenology. According to Walter theory, there are three components characterizing free will: (1) the ability to choose differently; (2) acting for (understandable) reasons, implying in no random behavior; (3) being the genuine source of one’s choices [8]. We acknowledge, though, that this topic is highly controversial, since there is no consensual definition of free will; actually, even its existence has been disputed [9]. Nevertheless, numerous studies have indicated that people’s beliefs in free will might be related to a range of behavioral features, including less cheating behavior [10], less aggression [11], better work performance [12], better self-control [13] and less conformity [14]. Thus, it seems relevant to investigate the relationship between people who experience behavioral disorders, such as OCD, and their free will beliefs.

Although there are few studies in this field, most are just conceptual discussions [7, 15, 16] or have important methodological short-comings, such as lack of information on appropriate case–control matching, evaluations that are cross-sectional, or even uncontrolled designs [17–21]. Also, these studies do not investigate the relationship between free will beliefs/experiences and symptoms’ severity. A recent review demonstrated the sparseness of the literature by identifying only three studies comparing free will beliefs between patients with psychiatric disorders (e.g. tics, addiction, and panic disorder) and healthy controls with using a valid tool (the Free Will And Determinism Scale (FAD) [22] and the Free Will and Determinism Scale (FWDS) [23]) [24]. This highlights the call for deeper investigations concerning free will and mental disorders, especially OCD.

To our knowledge, there is only one study that evaluated free will experiences quantitatively in an OCD sample. van Oudheusden and colleagues used an adapted version of the Symptomatology And Perceived Free will rating scale (SAPF) to investigate, cross-sectionally the perceptions of free will in OCD patients [17]. This tool, originally developed for patients with movements disorders [19], is based on the Walter theory of free will[8]. According to this study, patients with OCD have a diminished experience of free will when performing their compulsions that seems to be related to some clinical features, such as severity of symptoms, illness duration, quality of life and degree of insight [17].

Although these findings are of great interest, it is worthy to also investigate these experiences longitudinally and to compare the experiences of free will between patients and healthy controls. However, the use of the SAPF is limited to individuals showing a specific and/or “unusual” behavior and does not allow comparisons between individuals with vs. without a disorder. Therefore, to conduct a controlled study, the use of a scale measuring general free will beliefs is needed. Among the available tools [23, 25–29], the Free Will Inventory (FWI) stands out for not referring to any particular free will theory and also for including compatibilists’ views of free will [28].

In this study, we aimed (i) to compare the free will beliefs among OCD patients and healthy individuals; (ii) to evaluate whether the free will related cognitions (beliefs and experiences) change over the natural course of illness and (iii) to investigate whether the OCD patients free will related cognitions are able to predict changes in their symptoms’ severity. Two hypotheses were made. Firstly, we predicted that people with OCD would have a diminished belief in free will when compared to healthy controls. Secondly, we hypothesized that patients’ free will related cognitions would affect and be affected by treatment.

In this sense, we expected that the strength of patients’ beliefs or perceptions of free will over time would change and that those with greater free will scores would have a better treatment response. Of note, in the present study we have referred to the strength of people’s general belief in free will (as measured by the FWI) as “belief in free will”. When the free will exclusively concerned the patients’ repetitive behavior, we have referred to it as free will “perceptions” or “experiences” interchangeably (as measured by the SAPF). We have used the term “free will related cognitions” to refer either to free will experiences or beliefs.

## Methods

### Participants

The sample included a total of 60 OCD patients recruited consecutively at the OCD Clinic from the Obsessive, Compulsive, and Anxiety Spectrum Research Program Clinic at the Institute of Psychiatry of the Federal University of Rio de Janeiro. Inclusion criteria were as follows: (i) a primary diagnosis of OCD; (ii) age between 18 and above. People that were not able to read and fill out forms were excluded based on clinical grounds. Participants had their diagnosis confirmed by the Mini International Neuropsychiatric Interview (MINI) [30].

From initial 60 subjects, a subsample of 18 subjects with OCD was also age- and gender-matched post-hoc to a control sample of 18 healthy subjects recruited from the hospital’s administrative staff. All subjects were assessed for mental disorders with the MINI and did not fulfil criteria for any DSM-IV disorder. Table S1 of supplementary material summarizes the groups’ demographic and clinical characteristics. The local ethics committee approved the protocol and all patients provided written informed consent.

### Procedures

The participants were enrolled for the present study between April 2018 and October 2020. The totality of OCD subjects (*n* = 60) was assessed at baseline and re-evaluated after around one year of follow-up. Baseline data from 18 subjects with OCD were compared to age- and gender matched healthy controls. At baseline, demographic data, clinical characteristics, and free will related cognitions were assessed (described below).

All data from the 60 OCD subjects (except for sociodemographic information) was recollected after one year. Due to the COVID-19 outbreak, to limit the transmission of the new coronavirus in the healthcare setting, some participants with OCD had their baseline (*N* = 3) and/or follow-up (*N* = 32) data collected remotely through Research Electronic Data Capture (REDCap) hosted at the D’Or Institute for Research and Education (IDOR). Regarding healthy controls, only 4 of them had their data remotely collected.

Almost all patients (*N* = 58, 96.67%) were regularly treated with the maximum tolerated doses of serotonin reuptake inhibitors [fluoxetine (*N* = 26, 43.33%), clomipramine (*N* = 13, 21.67%), citalopram (*N* = 7, 11.67%), escitalopram (*N* = 7, 11.67%), paroxetine (*N* = 7, 11.67%), sertraline (*N* = 6, 10.00%) and fluvoxamine (*N* = 1, 1.67%)], with just one patient being treated with high dose venlafaxine and another one with high dose desvenlafaxine. Of note, clomipramine was added as an augmentation strategy to other selective serotonin reuptake inhibitors in 10 cases (16.67%).

### Measures

#### Demographics

Participants responded to a questionnaire that included information on age, gender, education, ethnicity, marital status and employment status.

#### Severity of symptoms

To assess severity of OCD symptoms, the self-report Yale-Brown Obsessive–Compulsive Scale (YBOCS) was administered. The YBOCS is the most widely used instrument to measure severity of OCD. It includes a total of 10 items that cover time, interference, anxiety or distress, resistance and control for obsessions and compulsions separately [31, 32]. Its scores vary from 0 to 40.

#### Psychological distress

Participants completed the Depression Anxiety Stress Scale [33, 34]. The DASS-21 contains 21 self-report items assessing depression, anxiety, and stress/tension symptoms. Respondents are asked to rate how much a specific statement applies to them during the past week (‘did not apply to me at all’ to ‘applied to me very much’, scored 0–3 respectively). The measurement of interest in this study was the total score, reflecting general psychological distress.

#### Free will beliefs

Beliefs in free will were assessed using FWI, a 29-item self-report measure divided into two parts, which can be given together or not [28, 35]. In the first one, participants rated the extent to which they agree with the statements from three 5-item subscales: Free Will (e.g., “People always have the ability to do otherwise”), Determinism (e.g., “Given the way things were at the Big Bang, there is only one way for everything to happen in the universe after that”), and Dualism and Non-reductionism (e.g., “Each person has a nonphysical essence that makes that person unique”). The second part has 14 statements regarding the relationships between beliefs in free will and people’s beliefs about causation, choice, the soul, predictability, responsibility, and punishment. Responses were based on a 7-point Likert-type scale ranging from 1 (strongly disagree) to 7 (strongly agree). For the purpose of this study, the strength of belief in free will was the measurement of interest. Therefore, only the three subscales of Part 1 were appraised.

#### Perceived free will

To assess the perceived free will, participants completed the SAPF, which was originally developed by Van der Salm et al. and further adapted by van Oudheusden et al. to explore the OCD free will perceptions [17, 19]. The 11 items of this questionnaire capture different aspects of the experience of free will, based on the conceptual framework by Walter [8].

His work is grounded by philosophical debates about free will and he points that it has three core elements [8], as previously described. The SAPF scale was developed to operationalize such insights in individuals with a repetitive behavior and these elements pointed by Walter were, respectively, termed as “alternative possibilities”, “intentionality” and “ownership” factors [17].

For the purposes of this study, the wording of the items was adapted to only consider the patient’s main compulsive behavior. Also, instead of using a visual analogue scale, responses were based on a 7-point Likert-type scale ranging from 0 (completely disagree) to 6 (completely agree). As proposed by van Oudheusden et al. we measured the scorings of the alternative possibilities (e.g., “You are able to suppress the compulsive behavior”), intentionality (e.g., “Your compulsive behavior is voluntary”) and ownership (e.g., “Your compulsive behavior is a part of you”) factors.

### Data analysis

Descriptive statistics were described in percentages, means and standard deviations, or medians and range (minimum–maximum). Normality of data was tested by Kolmogorov–Smirnov test. If the normality was rejected, then a non-parametric test was used. Quantitative variables were compared between two subgroups (18 OCD patients vs 18 age- and gender-matched healthy controls) using Mann–Whitney tests. To determine the relationships between continuous variables, a Spearman correlation coefficient was utilized in the total sample (60 OCD patients). In a second step, we conducted a generalized linear regression analysis with the final YBOCS score as the dependent variable and variables associated with these changes in the bivariate analysis as independent variables (baseline YBOCS score and SAPF subscores). Significance was set at 0.05. The analyses were carried out through SPSS version 23.0.

## Results

### Descriptive statistics

Sixty OCD patients were included and have completed all baseline assessments. The sample's mean age was 40.5 (SD 13.6) years, with 53.3% being females. The mean age at onset of OCD was 17.2 (SD 9.2) years, the mean OCD severity was 20.96 (SD 7.92), and the patients' main compulsive behavior included washing (28.3%), checking (16.7%), counting/mental rituals (13.3%), symmetry/ordering (1.7%), and other behaviors (40.0%). Patients were naturalistic followed up for 12.5 (SD 1.2) months on average. Since 13 patients (21.66%) dropped out (asked to not fill the endpoint forms or were not traceable), 47 patients have completed their participation in the longitudinal analysis. A detailed overview of the socio demographics and clinical features at baseline is provided in Table 1.

**Table 1:** Sample Demographics and Clinical Variables at Baseline

	OCD
	(*N* = 60)
**Age,** median (min–max)	37 (19–67)
**Gender,** N (%)	
Female	32 (53.3)
**Marital Status,** N (%)	
Single	38 (63.3)
Married	16 (26.7)
Divorced	5 (8.3)
Widowed	1 (1.7)
**Ethnicity,** N (%)	
Caucasian	37 (61.7)
Black	5 (8.3)
Asian	2 (3.3)
Other	16 (26.7)
**Education,** N (%)	
Up to 8 years	1 (1.7)
9 – 11 years	5 (8.3)
12 years or more	53 (88.3)
Non-informed	1 (1.7)
**Occupation,** N (%)	
Working	24 (40.0)
Unemployed	13 (21.7)
Retired due to disability	8 (13.3)
Retired due to contribution time	2 (3.3)
Housekeeping	1 (1.7)
Student	6 (10.0)
Sick leave	1 (1.7)
Never worked	1 (1.7)
Other	4 (6.6)
**OCD Severity,** mean (SD)	
YBOCS	20.96 (7.92)
**Current Psychiatric Comorbidity,** N (%)	
Major depression	19 (31.7)
Dysthymic disorder	1 (1.7)
Panic disorder	3 (5.0)
Agoraphobia	11 (18.3)
Social anxiety	9 (15.0)
PTSD	3 (5.0)
SUD	1 (1.7)
GAD	17 (28.3)

*OCD* Obsessive–compulsive disorder, *YBOCS* Yale Brown Obsessive–Compulsive Scale, *PTSD* Post-traumatic stress disorder, *SUD* Substance use disorder, *GAD* Generalized anxiety disorder

In relation to the psychometric characteristics of our main instruments of interest, the internal consistency and reliability, as measured with Cronbach’s alpha, were 0.815 (alternative possibilities factor), 0.053 (intentionality factor) and 0.806 (ownership factor) for SAPF and 0.660 (free will subscale), 0.613 (determinism subscale) and 0.724 (dualism subscale) for FWI.

### Comparison of FWI scores between OCD patients vs. healthy controls

The Free Will Inventory (FWI) was used to compare the strength of belief in free will between 18 patients with OCD and 18 age- and gender-matched healthy subjects but no significant difference in the beliefs regarding free will (U = 156.0; *p* = 0.864), determinism (U = 129.0; *p* = 0.308) or dualism (U = 162.0; *p* = 1.00) between these groups were found (see Table S1 in supplementary material).

### Correlations between free will related cognitions and clinical variables among OCD patients

To determine the relationships between variables measured at the baseline in the 60 subjects’ OCD sample, a Spearman correlation coefficient was utilized. As symptoms of psychological distress were significantly correlated with several free will related measures (see Table S2 in supplementary material), a partial correlation with the same variables, but controlling for DASS-21 total score, was performed (Table 2).

**Table 2:** Spearman’s partial correlation between clinical features and free will related measures at baseline, controlling for DASS-21 total score

		Age	Duration of illness	YBOCS total
FWI				
Free Will	ρ	0.098	0.002	0.007
	p-value	0.469	0.989	0.956
Determinism	ρ	-0.170	-0.317	0.107
	p-value	0.205	0.016*	0.429
Dualism	ρ	-0.021	-0.064	0.024
	p-value	0.878	0.636	0.861
SAPF				
Alternative Possibilities	ρ	-0.275	-0.085	-0.415
	p-value	0.038*	0.528	0.001*
Intentionality	ρ	-0.031	0.014	-0.155
	p-value	0.820	0.916	0.251
Ownership	ρ	0.016	0.010	0.538
	p-value	0.909	0.943	< 0.001*

*DASS* Depression Anxiety Stress Scales, *YBOCS* Yale-Brown Obsessive–Compulsive Scale, *FWI* Free Will Inventory, *SAPF* Symptomatology and Perceived Free Will Rating Scale

^*^ = *p* < 0.05

Our analysis showed that the SAPF’s alternative possibilities dimension was negatively correlated with both age and OCD severity of symptoms. We also found that the SAPF’s ownership construct correlated with severity of OCD symptoms (Table 2). In contrast, a negative correlation between the FWI’s belief in determinism and the duration of illness was found. Correlations between OCD dimensions and free will related cognitions are available in Supplemental Material (Table S3).

### Longitudinal assessments of free will related cognitions among OCD patients

We also investigated whether the OCD patients’ sample have changed their perception regarding the free will related cognitions after about one-year of a naturalistic follow-up. Our findings suggest that their beliefs and experiences tend to remain comparable to the initial ones (Table 3). Of note, we also found that the OCD sample showed a stabilization regarding its symptoms’ severity during this period (see Table S4 in supplementary material).

**Table 3:** Changes on free will related beliefs of OCD patients over their naturalistic follow up

	Baseline	Endpoint	Wilcoxon Signed Ranks
	Medians (min–max)	Medians (min–max)	
FWI			
Free will	25.0 (11–32)	25.0 (12–30)	Z = -0.778; *p* = 0.437
Determinism	16.5 (6–30)	17.0 (7–27)	Z = -0.206; *p* = 0.837
Dualism	26.0 (13–35)	26.0 (13–35)	Z = -0.145; *p* = 0.884
SAPF			
Alternative Possibilities	7.0 (0–21)	8.0 (0–23)	Z = -0.236; *p* = 0.814
Intentionality	9.0 (0–17)	9.0 (0–17)	Z = -1.695; *p* = 0.090
Ownership	6.0 (0–12)	6.0 (0–12)	Z = -0.512; p = 0.609

*OCD* Obsessive–Compulsive Disorder, *FWI* Free Will Inventory, *SAPF* Symptomatology And Perceived Free Will Rating Scale

### Free will related cognitions as predictors of OCD symptoms severity

Since the free will related measures have demonstrated some level of correlation with the initial severity of OCD symptoms, we performed a regression analysis to explore whether any of these measures were able to predict the YBOCS total score measured one year after the baseline assessments. The first analysis had the final YBOCS score as the dependent variable and the initial YBOCS score and FWI subscores as the independent variables.

The results indicated a statistically significant prediction model *F* (4,42) = 14.59; *p* < 0.001, with an R^2^ of 0.582 and an Adjusted R^2^ of 0.542. However, the evaluation of the main effects indicated that only the initial severity of OCD symptoms (YBOCS total at baseline) was statistically significant (Table 4).

**Table 4:** Multiple regression predicting YBOCS scores using free will related measures as independent variables

	β	t	*P*
FWI			
YBOCS total (baseline)	0.706	6.821	< 0.001*
Free will	-0.166	-1.445	0.156
Determinism	0.192	1.559	0.127
Dualism	-0.013	-0.112	0.911
SAPF			
YBOCS total (baseline)	0.513	3.769	0.001*
Alternative possibilities	0.011	0.103	0.919
Intentionality	-0.143	-1.318	0.195
Ownership	0.315	2.415	0.020*

*YBOCS* Yale-Brown Obsessive–Compulsive Scale, *FWI* Free Will Inventory, *SAPF* Symptomatology and Perceived Free Will Rating Scale

^*^ = *p* < 0.05

The second analysis also considered the final YBOCS score as the dependent variable, but also the SAPF subscores as potential predictors. Our results indicated a statistically significant prediction model *F* (4,41) = 16.106; *p* < 0.001, with an R^2^ of 0.611 and an Adjusted R^2^ of 0.573. The evaluation of the main effects indicated that both the initial severity of OCD symptoms (YBOCS total at baseline) and the ownership factor of the SAPF were statistically significant. Results are presented in Table 4.

## Discussion

In this study, we compared free will beliefs between patients with OCD and healthy subjects and assessed whether free will related cognitions (beliefs or experiences) were associated with differential outcomes obtained with a traditional therapeutic approach to OCD (high dose SRIs). We are not aware of any previous study that investigated whether free will related cognitions predict long term treatment outcomes of OCD patients. Our findings suggest that, regardless of the general beliefs in free will that OCD patients have, the experiences of free will concerning their repetitive behaviors seem to correlate with age and severity of OCD. The latter, along with the basal severity of symptoms, predicted symptom severity outcome of individuals with OCD. In addition, patients with a longer duration of illness tend to have a lower strength of belief in determinism.

As previously mentioned, OCD patients usually report a sense of being compelled to perform a particular act [36]. However, we found no significant differences in the free will beliefs between patients with OCD and healthy individuals, suggesting that having compulsive behaviors do not affect – or are not affected by – these general beliefs. Albeit we acknowledge that a comparison with a larger sample size would be desirable, our findings are in line with a study that investigated free will beliefs among another compulsive spectrum disorder and healthy subjects. van der Salm and colleagues found that patients with tics and controls do not differ in terms of general beliefs in free will [19]. Therefore, it seems that OCD patients’ general beliefs are not affected by the experience of having a compulsive behavior.

This perspective is endorsed by the findings presented in Table S2 of the supplementary material, which shows no correlation between the FWI subscales and the SAPF factors. However, our results suggest that some of these cognitions are correlated with patients’ clinical features. We found that the longer the duration of illness, the lower the strength of the belief in determinism. As OCD tends to have a chronic course [37], one potential interpretation is that patients’ symptoms experiences might influence their belief in determinism overtime. In other words, patients get the ability to reflect upon motivations of their OCD behaviors, which are then reviewed and contrasted to their past acts, decisions, and life events. As a result, their strength of belief in determinism might decrease, without necessarily changing the free will perceptions they have about their compulsive behaviors. Of note, van Oudheusden et al. found that a longer duration of illness was associated with a lower “alternative possibilities” factor scoring [17].

It is important to emphasize that the post-hoc explanation describe above does not concern aging, since age has no correlation with any FWI subscores. In terms of the SAPF, it seems that younger patients tend to have a higher perceived ability to choose differently, as indicated by the correlation between age and the SAPF alternative possibilities factor. We can only speculate on the potential interpretation of these findings. For instance, cognitive factors, including age-related cognitive rigidity [38], may lead patients to perceive themselves as being less able to choose alternatives other than engaging in their compulsive behavior. This phenomenon may be akin to age related decreases in self-efficacy [39]. In contrast, younger patients may not perceive their behaviors as rigid or habitual as older individuals.

In line with van Oudheusden et al. findings [17], our results also indicate that patients with greater severity of symptoms tend to feel that they do not have different behavioral options besides performing that specific compulsive act. This outcome is not so surprising, considering the OCD psychopathology itself, where patients feel driven to perform habitual behaviors in response to an internal distress [40]. Therefore, the more unbearable this state, the greater the perception that performing a specific behavior is the only way to cope. In addition, the above mentioned authors also suggest that the correlation between the experience of alternative possibilities and the severity of symptoms reflect an overlap between this SAPF factor and some of the YBOCS items [17].

Differently from van Oudheusden et al. [17], however, we found a significant relationship between the symptoms severity and the ownership factor. Patients with a higher YBOCS score exhibit a stronger sense of ownership of their behaviors. It is difficult to explain this apparently contradictory finding. However, it is possible that the ownership factor is associated with an illusory perception of free will in OCD patients. An alternative explanation, which was raised by van Oudheusden et al., is that these factor items are measuring OCD related egosyntonicity instead of free will [17]. Interestingly, van Oudheusden et al. also found that higher scores of ownership were associated with poorer insight according to the Overvalued Ideas Scale [17, 41]. It is known that a poorer insight on OCD relates to egosyntonic symptoms [42]. Therefore, it seems plausible that these patients might have a higher sense of ownership, since they perceive their symptoms as part of their identity.

Another related topic to be addressed is the evidence that OCD patients with poorer insight tend to present a worse outcome [43]. In line with these findings, our results suggest that a greater sense of ownership is related to a worse long-term outcome independently of baseline YBOCS scores. As expected, baseline YBOCS also predicted worse response. Our model suggests that the degree to which patients experience their compulsions as their own behavior can, indeed, modify the course of their illness. Thus, rather than increasing free will, the sense of ownership might actually reflect greater severity and, therefore, decreased free will toward compulsive behaviors.

These findings may have implications for clinical practice, since it is therapeutically useful to assume that patients can exercise some free will and choose to work toward a better and more fulfilling lifestyle [44]. For instance, cognitive behavior therapy, one of the gold standard treatments for OCD, encourages patients to abstain from behaviors that neutralize obsessions [45], thus highlighting the importance of free will in the therapeutic process. Therefore, therapists might want to work with clients to readjust the sense of ownership of their behaviors, which could be redirected towards living a more meaningful life.

Our study has several limitations. For instance, a larger sample of healthy subjects would allow a more robust comparison regarding free will beliefs with OCD patients. However, as previously stated, our findings are in consonance with data from another study comparing individuals with an OCD-related disorder (Tic disorder) with healthy controls [19]. Another potential limitation is the fact that 21.7% of the OCD participants were not re-assessed at the endpoint. Nonetheless, these rates are within acceptable ranges and comparable to other similar naturalistic studies [46–48]. In addition, we cannot exclude the possibility that the lack of difference between endpoint and baseline scores on the YBOCS is related to the inclusion of patients who were already on the long-term treatment and therefore relatively stable. Likewise, it was not surprising that the baseline YBOCS has strongly predicted the symptoms severity at follow-up. As for the original SAPF scale [17], internal consistency for the intentionality factor was low, raising issues regarding the homogeneity of this construct. Finally, although this study is the first to evaluate the free will perceptions of OCD patients longitudinally, the research in this field would benefit from longer follow-up assessments.

## Conclusions

The results of this study provide the first data about the longitudinal evaluation of free will related cognitions in patients with OCD. Our findings suggest that (i) patients with OCD and healthy controls do not differ in terms of their beliefs in free will; (ii) patients with a longer duration of illness tend to have a lower strength of belief in determinism; (iii) OCD patients with a greater severity of symptoms or higher age exhibit a diminished perception of free will and (iv) the experience of being the owner of the compulsions, along with the basal severity of symptoms, can predict a worse prognosis of OCD.

## Supplementary Information

**Additional file 1:** **Table S1**. Comparisons between demographics and clinical variables of OCD patients vs. healthy controls. **Table S2.** Spearman’s correlation between clinical features and free will related measures at baseline.** Table S3.** Spearman’s correlation between OCI-R and free will related measures variables at baseline.** Table S4.** Changes on OCD severity.

## Data Availability

The datasets generated and analysed during the current study are not publicly available due to limitations of ethical approval involving the participant data and anonymity but are available from the corresponding author on reasonable request.

## Abbreviations

DASS: Depression Anxiety Stress Scale
DSM: Diagnostic and Statistical Manual of Mental Disorders
FAD: Free Will And Determinism Scale
FWDS: Free Will And Determinism Scale
FWI: Free Will Inventory
MINI: Mini International Neuropsychiatric Interview
OCD: Obsessive–compulsive disorder
REDCap: Research Electronic Data Capture
SAPF: Symptomatology And Perceived Free will rating scale
YBOCS: Yale-Brown Obsessive–Compulsive Scale
