# Supplementary material for: A longitudinal evaluation of free will related cognitions in obsessive–compulsive disorder
Source: BMC Psychiatry. 2022 Jul 13;22:463. doi: 10.1186/s12888-022-04108-6 (PMC9277897; doi:10.1186/s12888-022-04108-6)
Supplement: Supplementary file 1 — Additional file 1: Table S1. Comparisons between demographics and clinical variables of OCD patients vs. healthy controls. Table S2. Spearman’s correlation between clinical features and free will related measures at baseline. Table S3. Spearman’s correlation between OCI-R and free will related measures variables at baseline. Table S4. Changes on OCD severity. [file 12888_2022_4108_MOESM1_ESM.docx]

**Supplementary material**

**Table S1**

|  | OCD |  | Controls |  | Statistics |
| --- | --- | --- | --- | --- | --- |
|  |  |  |  |  |  |
|  | (N = 18) |  | (N = 18) |  |  |
|  |  |  |  |  |  |
| **Age,** median (min-max) | 30 (21-55) |  | 30 (21-51) |  | U = 154.5; p = 0.815 |
| **Gender,** N (%)  Female | 14 (77.8) |  | 14 (77.8) |  | *X*^2^(1) = 0.0; p = 0.655 |
| **Marital Status,** N (%)  Single  Married  Widowed | 13 (72.2)  4 (22.2)  1 (5.6) |  | 12 (66.7)  6 (33.3)  0 (0.0) |  | *X*^2^(2) = 1.394; p = 0.711 |
| **Ethnicity,** N (%)  Caucasian  Black  Asian  Other | 7 (38.9)  4 (22.2)  2 (11.1)  5 (27.8) |  | 12 (70.6)  2 (11.8)  0 (0.0)  3 (17.6) |  | *X*^2^(4) = 4.667; p = 0.294 |
| **Education,** N (%)  Up to 8 years  9 – 11 years  12 years or more | 0 (0.0)  0 (0.0)  18 (100.0) |  | 1 (5.6)  1 (5.6)  16 (88.8) |  | *X*^2^(2) = 1.985; p = 0.486 |
| **Occupation,** N (%)  Working  Unemployed  Retired due to disability  Student  Other | 7 (38.9)  7 (38.9)  1 (5.6)  2 (11.1)  1 (5.6) |  | 15 (83.3)  0 (0.0)  0 (0.0)  3 (16.7)  0 (0.0) |  | *X*^2^(4) = 12.290; p = 0.003* |
| **Current Psychiatric Comorbidity,** N (%)  Major depression  Panic disorder  Agoraphobia  Social anxiety  PTSD  GAD | 7 (38.9)  1 (5.6)  7 (38.9)  3 (16.7)  2 (11.1)  5 (27.8) |  | NA  NA  NA  NA  NA  NA |  |  |

Comparisons between demographics and clinical variables of OCD patients vs. healthy controls

Footnote: OCD = Obsessive-Compulsive Disorder; PTSD = Post-Traumatic Stress Disorder; GAD = Generalized Anxiety Disorder; NA = Not applicable; * = p < 0.05

**Table S2**

Spearman’s correlation between clinical features and free will related measures at baseline

|  |  | Age | Duration of illness | YBOCS total | DASS-21 | Free will | Determinism | Dualism |
| --- | --- | --- | --- | --- | --- | --- | --- | --- |
| FWI |  |  |  |  |  |  |  |  |
| Free Will | ρ | 0.121 | 0.031 | 0.024 | 0.033 | - | 0.431 | 0.269 |
|  | p-value | 0.357 | 0.815 | 0.853 | 0.802 | - | 0.001* | 0.038* |
| Determinism | ρ | -0.098 | -0.221 | 0.191 | 0.261 | - | - | 0.408 |
|  | p-value | 0.455 | 0.092 | 0.144 | 0.045* | - | - | 0.001* |
| Dualism | ρ | 0.023 | -0.005 | 0.106 | 0.218 | - | - | - |
|  | p-value | 0.860 | 0.971 | 0.421 | 0.098 | - | - | - |
| SAPF |  |  |  |  |  |  |  |  |
| Alternative Possibilities | ρ | -0.296 | -0.101 | -0.414 | -0.097 | 0.097 | -0.032 | 0.080 |
|  | p-value | 0.023* | 0.452 | 0.001* | 0.466 | 0.465 | 0.812 | 0.546 |
| Intentionality | ρ | -0.059 | 0.012 | -0.150 | -0.020 | -0.067 | -0.164 | -0.032 |
|  | p-value | 0.660 | 0.928 | 0.256 | 0.879 | 0.615 | 0.215 | 0.807 |
| Ownership | ρ | 0.029 | 0.079 | 0.592 | 0.350 | 0.029 | 0.188 | 0.180 |
|  | p-value | 0.829 | 0.554 | < 0.001* | 0.007* | 0.829 | 0.155 | 0.173 |

Footnote: YBOCS = Yale-Brown Obsessive-Compulsive Scale; DASS = Depression Anxiety Stress Scales; FWI = Free Will Inventory; SAPF = Symptomatology and Perceived Free Will Rating Scale; * = p < 0.05

**Table S3**

Spearman’s correlation between OCI-R and free will related measures variables at baseline

|  |  | Washing | Checking | Ordering | Obsessing | Hoarding | Neutralizing |
| --- | --- | --- | --- | --- | --- | --- | --- |
| FWI |  |  |  |  |  |  |  |
| Free Will | ρ | -0.076 | 0.199 | 0.064 | -0.090 | 0.249 | 0.027 |
|  | p-value | 0.563 | 0.127 | 0.627 | 0.492 | 0.055 | 0.841 |
| Determinism | ρ | -0.121 | 0.187 | 0.131 | 0.256 | 0.276 | 0.186 |
|  | p-value | 0.356 | 0.153 | 0.318 | 0.048* | 0.033* | 0.154 |
| Dualism | ρ | -0.141 | -0.197 | -0.053 | -0.066 | 0.035 | 0.204 |
|  | p-value | 0.281 | 0.131 | 0.686 | 0.618 | 0.789 | 0.118 |
| SAPF |  |  |  |  |  |  |  |
| Alternative Possibilities | ρ | -0.189 | -0.114 | -0.207 | -0.313 | -0.366 | -0.152 |
|  | p-value | 0.152 | 0.390 | 0.116 | 0.016 | 0.004* | 0.249 |
| Intentionality | ρ | 0.120 | 0.082 | -0.031 | -0.165 | -0.032 | -0.219 |
|  | p-value | 0.365 | 0.536 | 0.818 | 0.213 | 0.808 | 0.095 |
| Ownership | ρ | 0.292 | 0.346 | 0.347 | 0.397 | 0.234 | 0.355 |
|  | p-value | 0.025* | 0.007* | 0.007* | 0.002* | 0.074 | 0.006* |

Footnote: OCI-R = Obsessive-Compulsive – Revised; FWI = Free Will Inventory; SAPF = Symptomatology and Perceived Free Will Rating Scale; * = p < 0.05

**Table S4**

Changes on OCD severity

|  | Baseline |  | Endpoint |  | Statistics |
| --- | --- | --- | --- | --- | --- |
|  | Mean (SD) |  | Mean (SD) |  |  |
|  |  |  |  |  |  |
| YBOCS obsessions | 10.49 (3.9) |  | 10.0 (4.22) |  | t(46) = 1.08; p = 0.284 |
|  |  |  |  |  |  |
| YBOCS compulsions | 10.47 (4.32) |  | 9.49 (4.96) |  | t(46) = 1.88; p = 0.066 |
|  |  |  |  |  |  |
| YBOCS total | 20.96 (7.92) |  | 19.49 (8.55) |  | t(46) = 1.69; p = 0.098 |
|  |  |  |  |  |  |

Footnote: OCD = Obsessive-Compulsive Disorder; YBOCS = Yale Brown Obsessive-Compulsive Scale
